# Clinical characteristics and laboratory parameters of deep venous thrombosis after SARS-CoV-2 infection in a non-hospital setting – a single-center retrospective study

**DOI:** 10.3325/cmj.2022.63.448

**Published:** 2022-10

**Authors:** Sara Sablić, Sanja Lovrić Kojundžić, Danijela Budimir Mršić, Dragan Dragičević, Maja Marinović Guić, Ivan Kraljević, Antonela Matana

**Affiliations:** 1 Clinical Department of Diagnostic and Interventional Radiology, University Hospital Split, Split, Croatia; 2 University of Split School of Medicine, Split, Croatia; 3 University Department of Health Studies, University of Split, Split, Croatia

## Abstract

**Aim:**

To assess the differences in clinical and laboratory parameters of non-hospitalized patients with deep venous thrombosis (DVT) according to the SARS-CoV-2 status.

**Methods:**

We retrospectively reviewed demographic, clinical, laboratory, and ultrasound data of adult patients admitted to the Emergency Department of University Hospital Split between March 2020 and January 2021. Patients were classified into three groups: recent COVID-19 (<1 month), non-recent COVID-19 (1 to 12 months), and non-COVID-19.

**Results:**

Fifty (47.2%) of 106 patients had a history of SARS-CoV-2 infection (23 patients in the recent COVID-19 and 27 in non-recent COVID-19 group). The three groups did not significantly differ in demographic and clinical parameters, including the location of deep venous thrombosis. The recent COVID-19 group had significantly higher neutrophils and CRP levels, and significantly lower prothrombin than the other two groups.

**Conclusion:**

Our results confirm the role of elevated inflammatory and coagulation response in DVT development in the first month after the infection, but not in non-recent COVID-19 or non-COVID-19 patients.

Hospitalized patients infected with coronavirus disease 2019 (COVID-19) were reported to suffer from a hypercoagulable state, characterized by changes in coagulation tests (elevated D-dimer, prolonged prothrombin time [PT], activated partial thromboplastin time (aPTT), and/or low fibrinogen levels) (1-3) and higher levels of C-reactive protein (CRP), lactate dehydrogenase (LDH), and transaminases (4). These patients, especially those admitted to the intensive care unit (ICU), also showed a relatively high incidence of deep venous thrombosis (DVT) (1), with predominant involvement of the distal veins of the leg (1,5,6). Male sex, high body mass index, hypertension, and ICU admission were risk factors for developing DVT (1-3,7,8). Finally, the majority of the DVT events occurred within three weeks after a positive COVID-19 test, and DVT incidence returned to baseline approximately 40 days after testing (9).

In contrast to thromboembolic events in hospitalized COVID-19 patients, DVT occurring in a non-hospital setting has been rarely investigated. Possible reasons might be relatively low DVT incidence in this population and difficulties in monitoring the disease course in the non-hospital setting. The present study compared the clinical and laboratory characteristics of DVT in recent COVID-19, non-recent COVID-19, and non-COVID-19 patients who were admitted to our emergency department (ED).

## Patients and methods

Clinical radiologists with long experience in vascular sonography retrospectively reviewed the medical records of patients suspected of having DVT who were admitted to the Emergency Internal Medicine Department of the University Hospital Split between March 2020 and January 2021. DVT was diagnosed based on ultrasonography performed with a linear 7.5 MHZ probe (LOGIQ S8, Medical Systems Inc., Milwaukee, WI, USA) in real-time B-mode using Color-Doppler in transverse and longitudinal scans. Both proximal and distal deep venous system were evaluated. The criteria for DVT diagnosis were a lack of vein compressibility or direct identification of an endoluminal thrombus. Localization of the DVT was categorized into proximal leg thrombosis, distal leg thrombosis, or both. Proximal leg thrombosis included thrombosis of the deep veins of the upper leg and popliteal vein, while distal thrombosis included DVT of the deep veins of the lower leg.

**Figure 1:**
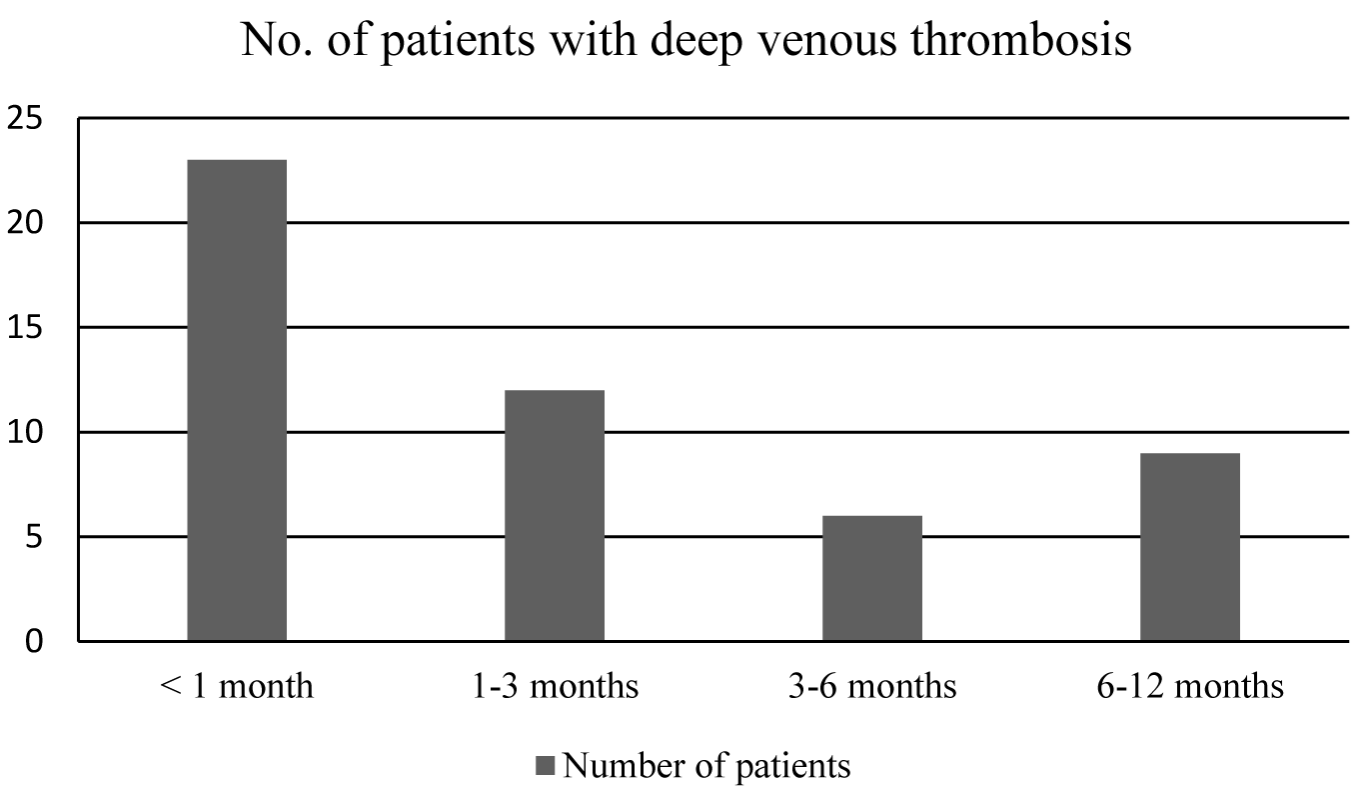
The number of patients with recent and non-recent coronavirus disease 2019 (COVID-19) and deep venous thrombosis according to the time interval since COVID-19 infection.

All patients were tested for an acute SARS-CoV-2 infection (rapid antigen test) in the ED as a standard protocol. Patients with confirmed DVT at admission were included in the study and were divided into three groups based on SARS-CoV-2 infection status: a group with an active SARS-CoV-2 infection or positive SARS-CoV-2 test within the last month (recent COVID-19 group); a group with no active infection or positive test within last one month but with a history of SARS-CoV-2 infection during the last 12 months (non-recent COVID-19 group) (Figure 1); and a group with no history of SARS-CoV-2 infection (control group) and a negative rapid antigen test. None of these patients were SARS-Cov-2 positive at ED admission.

Patients underwent standard laboratory testing including the assessment of coagulation parameters. Data on cardiovascular risk factors, comorbidities, and laboratory tests were collected from electronic medical records in the hospital information system. The study complied with the 1975 Declaration of Helsinki and was approved by the Ethics Committee of the University Hospital Split.

### Statistical analysis

The Kolmogorov-Smirnov test was used for normality testing. Data are presented as median (Q1-Q3). Differences in categorical variables were assessed with a χ^2^ test and a Fisher exact test. Differences in continuous variables were assessed with a Mann-Whitney test and a Kruskal-Wallis test. The significance level was set at *P* < 0.05. Data were analyzed with IBM SPSS, version 28.0 (IBM Corp., Armonk, NY, USA).

## Results

The study enrolled 106 consecutive patients: 23 patients with recent COVID-19 (median age 60 years); 27 patients with non-recent COVID-19 (median age 64 years); and 56 patients in the non-COVID-19 group (median age of 68 years). None of the patients with a history of SARS-CoV-2 infection required mechanical ventilation/oxygen therapy. All of them were treated at home during the infection. Only five patients (10%) underwent a chest x-ray during the acute phase of the illness, which showed bilateral interstitial pneumonia. The three groups did not differ in age, sex, comorbidities, or thromboprophylactic therapy (Table 1).

**Table 1:** Demographic and clinical characteristics of patients with deep venous thrombosis

	Recent COVID-19	Non-recent- COVID-19	Non- COVID-19	P (Fisher exact test)
N (%)	23 (21.7)	27 (25.4)	56 (52.8)	
Age (years), median (Q1-Q3)	60 (49-74)	64 (59-74)	68 (59-75)	0.411
Male patients	11 (47.8)	11 (40.7)	28 (50)	0.904
Hypertension	7 (30.4)	13 (48)	24 (48)	0.459
Diabetes mellitus	4 (17.4)	3 (11.1)	7 (12.5)	0.803
Cardiovascular diseases	9 (39.1)	6 (22.2)	12 (21.4)	0.246
Cerebrovascular diseases	0 (0)	0 (0)	6 (10.7)	0.077
Malignancy	2 (8)	5 (18.5)	11 (19.6)	0.544
Smoking	0 (0)	1 (0.3)	7 (12.5)	0.117
Thromboprophylaxis	6 (26)	8 (29.6)	13 (23.2)	0.836

The recent COVID-19 group had significantly higher levels of neutrophils (*P* = 0.008), CRP (*P* = 0.009), and LDH (*P* = 0.009), while white blood cell count was similar among the groups (*P* = 0.438). The recent COVID-19 group also had significantly lower PT (*P* = 0.045) and significantly higher D-dimer (*P* = 0.038) compared with other groups (Table 2).

**Table 2:** Laboratory parameters of patients with deep venous thrombosis

	Recent COVID-19	Non-recent COVID-19	Non- COVID-19	P (Kruskall Wallis test)
White blood cells ([10^9^]/L)	10.55 (7.52-13.5)	8.25 (6.9-12.42)	9 (7.7-10.4)	0.438
Neutrophils (%)	74.2 (67.9-89.6)	64.60 (60.92-73.22)	66 (63-77.8)	0.008
Prothrombin time	1.12 (0.96-1.16)	1.165 (1.13-1.28)	1.23 (1.06-1.36)	0.045
Activated partial thromboplastin time (s)	22.6 (21.1-23.75)	23.3 (20.47-24.92)	21.3 (19.97-23.25)	0.103
D-dimer (mg/L)	12.11 (5.49-25.61)	5.08 (3.11-12.32)	4.36 (1.86-12.85)	0.038
Lactate dehydrogenase (U/L)	261.5 (227.5-333.25)	258 (218-353)	213 (171.5-262.5)	0.009
C-reactive protein	48.3 (15.45-88.95)	16.2 (6.9-28.72)	14.5 (6.33-53.47)	0.009

*data are presented as median (Q1-Q3).

**Figure 2:**
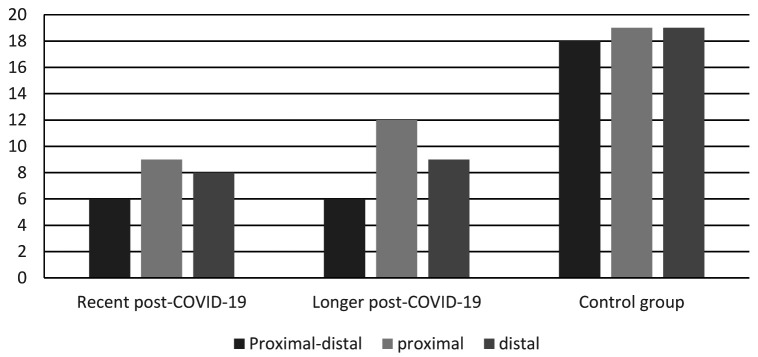
Localization of deep venous thrombosis in recent coronavirus disease 2019 (COVID-19), non-recent COVID-19, and control group (*P* = 0.871).

Patients with recent COVID-19 had significantly higher neutrophils (*P* = 0.003) and CRP (*P* = 0.007) and significantly lower PT (*P* = 0.033) than patients with non-recent COVID-19. These two groups did not significantly differ in other laboratory parameters. Localization of DVT was similarly distributed between the groups (χ^2^ = 1.24, df 4, *P* = 0.871) (Figure 2).

## Discussion

Our results confirmed the role of elevated inflammatory and coagulation response in DVT development in the first month after the infection, but not in non-recent COVID-19 or non-COVID-19 patients. We enrolled patients whose SARS-CoV-2 infection presentation was generally mild and who did not require hospital admission and/or oxygen therapy. The majority of patients did not receive thromboprophylaxis. We showed that this population was also in danger of developing DVT while recovering from COVID-19 at home. Most of the published research involved hospitalized COVID-19 patients (ICU or ward patients) who had abundant pulmonary infiltrates and received thromboprophylactic therapy because of the known hypercoagulable state caused by COVID-19.

In our study, all the examined groups had similar comorbidities and risk factors. DVT in the recent COVID-19 group was associated with significantly higher neutrophils, D-dimers, LDH and CRP, compared with the control group, and with significantly higher neutrophils and CRP compared with non-recent COVID-19 group. Of coagulation parameters, the recent COVID-19 group had lower prothrombin time. These findings contribute to the thesis of viral hyperinflammatory response and hypercoagulable state playing important roles in the pathogenesis of thrombotic events during acute infection and early post-COVID-19 period.

Recent research conducted on hospitalized COVID-19 patients who underwent early ultrasound investigation showed that the most common localization of DVT was distal veins of the leg (1-3,10,11). These patients, even despite regular thromboprophylactic therapy, had a relatively high occurrence of DVT (6,11). In contrast, our study showed no differences in DVT localization between the groups, a finding that may imply that lower DVT, which is the most common type of DVT, could remain unrecognized in the non-hospital setting. In addition, not receiving thromboprophylactic therapy, which was the case in 70% of patients, could contribute to the development of both upper and lower extremity thrombosis at a similar percentage.

Finally, our study showed a similar number of patients with DVT in the recent COVID-19 period (up to 1 month) (n = 23) and in the non-recent COVID-19 period (n = 27). Significantly more COVID-19-related DVTs occurred within the first month, which is in line with other studies (3,10,12) that showed a similar peak of incidence during the highest viral inflammatory response.

The study limitations included single-center design and a relatively small number of patients. In addition, the retrospective design prevented us from collecting relevant laboratory data for some of the patients (fibrinogen was analyzed in only 4 patients).

In conclusion, inflammatory and coagulation parameters of post-COVID-19 thrombosis may trigger the onset of DVT. Patients with mild COVID-19 symptoms require clinical and laboratory follow-up. Larger studies are needed to identify the comorbidities and laboratory parameters indicative of post COVID-19 DVT, as well as to identify candidates for thromboprophylactic therapy in the non-hospital setting.
